# Twin Pregnancy Obtention of Patient with Nonmosaic Klinefelter’s Syndrome and His Wife with Chromosome 9 Inversion by ICSI Treatment

**Published:** 2013-07-31

**Authors:** Changjun Zhang, Haiying Peng, Yueyue Hu

**Affiliations:** The Reproductive Research Center, Renmin Hospital, Hubei university of Medicine, Shiyan, China

**Keywords:** FISH, Klinefelter’s Syndrome, Infertility, ICSI, Chromosome Inversion

## Abstract

A 24-year-old man was diagnosed with klinefelter’s syndrome (KS) and his wife was found to have an inversion on chromosome 9-46, XX, inv (9) (p11q21)- because of infertility. Intracytoplasmic sperm injection (ICSI) was performed for fertilization after fluorescence in-situ hybridization (FISH) was used to analyze the aneuploidy rate of the X and Y chromosomes of the ejaculated sperms of the patient, and 99 sperms were haploid among 100 sperms that were to be analyzed. A twin pregnancy was achieved. The chromosomes of the two fetuses were identified as 46, XY and 46, XY, inv (9)(p11q21) after a prenatal diagnosis at 18 weeks gestation. Two healthy twins were born through caesarean section at 32 weeks gestation because of premature rupture of membranes (PROM).

## Introduction

Klinefelter’s syndrome (KS) is the most frequent sex chromosomal abnormality and is usually pathologically characterized by spermatogenic dysfunction (1). It includes two kinds of karyotypes: mosaic and non-mosaic type where ninety percent of men with KS are non-mosaic type (2-3). The clinical symptoms of KS include no or reduced sperm production and severe asthenozoospermia, although such patients can still acquire offspring by ICSI (4-7).

Pericentric inversion of chromosome 9 is one of the frequent chromosomal rearrangements, considered a normal variant with a prevalence of 1-3% (8-10). Although it may not be correlated with abnormal phenotypes, there were some reports indicating that it was associated with infertility (8), congenital heart disease (11), dysmorphic features and other congenital anomalies. It was suggested that there might be loss or suppression of euchromatin chromosome region by an inversion event, therefore, further detailed chromosomal break point study may help us to better understand its mechanisms and clinical significance (12).

Here, we report a case of KS, in which fluorescence in-situ hybridization (FISH) was used to analyze the aneuploidy rate of the ejaculated sperm of the patient, while identifying an inversion in chromosome 9 in his wife. For the first time, intracytoplasmic sperm injection (ICSI) was performed to treat the patient, who fathered a twin pregnancy successfully.

## Case Report

A married couple (a 24-year-old man and a 22-year-old woman) were treated at our center for a diagnosis of primary infertility. A physical examination found the following in the man: height, 178 cm; span height, 178 cm; sitting height, 98 cm; weight, 79 kg; a history of mumps; normal secondary sexual characteristics; and a negative response to anti-sperm antibody.

**Fig 1:**
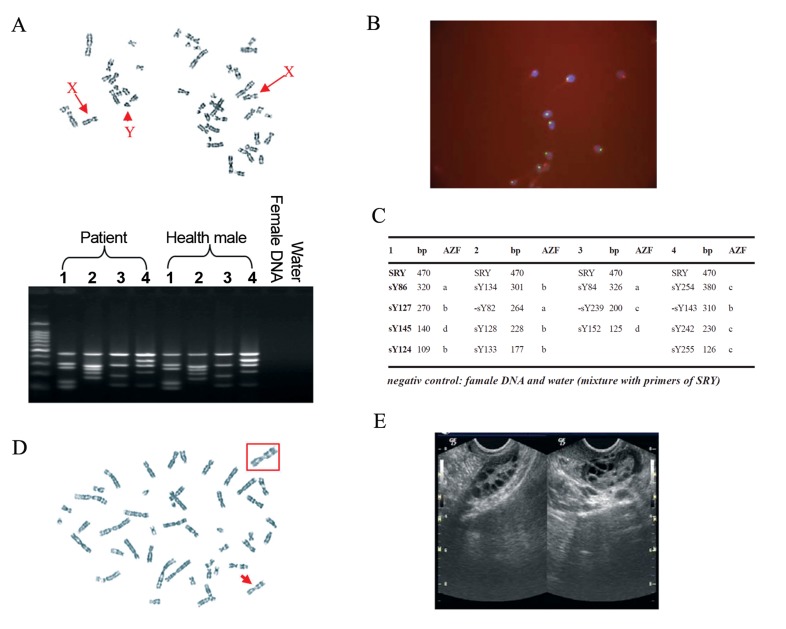
The clinical details of the couple. A. Karyotype of the husband (47, XXY). B. The sperm were analyzed by FISH (×1000). C. The result indicated the husband without AZF deletion after multiplex PCR analysis. D. karyotype of the wife [46, XX, inv(9) (p11q21), and E. TVS found multiple immature follicles in her both of ovaries

Results of an external genitalia examination were normal for pubic hair, penis length (8 cm), bilateral epididymitis, vas deferens, and prostate, but no antheridiogen varicose veins and bilateral testes of a small size (~5 ml). A semen examination revealed severe asthenozoospermia, of which the sperm showed a volume of 0.8 ml and a density of 0.7×10^6^/ ml (World Health Organization standard, 2010). His peripheral blood karyotype was non-mosaic 47, XXY after we analyzed 200 peripheral lymphocytes. The karyotyping results of 100 oral exfoliated cells were also analyzed and the diagnosis was confirmed. No AZF deletion was found after multiplex PCR analysis. Based on a previously published method (13), FISH was used to evaluate the sperms and found that the hybridization efficiency was 99% in 100 analyzed sperms, all of the hybridized sperms were haploid (47 normal X sperm and 52 normal Y sperm, the ratio of X and Y sperm was 0.9:1) (Fig 1A-C). His hormone levels were as follows: folliclestimulating hormone (FSH), 26.40 mIU/mL; luteinizing hormone (LH), 9.57 mIU/mL; estrogen (E2), 26.00 pg/mL; prolactin (PRL), 20.7 ng/mL; testosterone (testo), 2.18 ng/mL and progesterone (P), 19.37 ng/mL. Based on these results, the patient was diagnosed with KS.

His wife had a menstrual cycle of 4/37 and mild dysmenorrhea, normal vulva and vagina, smooth cervix without masts or lacerations, a normal-sized posterior uterus with medium texture, no active tenderness; and normal bilateral attachments with no lesions or tenderness. The basal endocrine index was as follows: FSH, 6.8 mIU/mL; LH, 5.2 mIU/mL; E2, 19.6 pg/mL; thyroid hormone (T1), 17.7 ng/dL; PRL, 20.7 ng/mL; testo, 0.94 ng/mL and P, 0.41 ng/mL. Multiple immature follicles were observed in both of ovaries by transvaginal ultrasound (TVS) (Fig 1E), thus, she was diagnosed with polycystic ovary syndrome (PCOS).

Karyotype examination of 200 peripheral blood cells showed pericentric inversion of one chromosome 9, with the breakpoint in the short arm at 9p11 and in the distal region of the long arm at 9q21 (Fig 1D). The woman’s immediate family members had no history of adverse pregnancies and there were no genetic abnormalities in her family history.

Before the couple agreed to ‘assisted reproductive treatment, they received detailed genetic counseling and chose the ICSI treatment. Furthermore, the patient and his wife rejected a preimplantation genetic diagnosis, but were willing to undergo second trimester prenatal diagnoses. The wife received a short controlled ovarian hyperstimulation (COH), and the protocol was as follows: 1. at menstrual day 3, an intramuscular injection of Decapeptyl (gonadotropin- releasing hormone agonist, GnRHa) at a dose of 0.65 mg was carried for down-regulation; 2. from day 5, Gonal-F (recombinant FSH, rFSH) was continually used at a dose of 150 IU/d to promote ovulation; 3. at day 9, the dose of Gonal-F was reduced to 75 IU/d, at the same time, Luveris (recombinant LH: rLH) was injected at a dose of 75 IU/d; 4. serial ultrasound examinations and the evaluation of serum E2, LH, and P levels were used to monitor the follicular maturation. Pregnyl (hCG) was employed for ovulation induction at a dose of 10000 IU IM when at least two follicles achieved a mean diameter of 18 mm; 5. oocyte aspiration was performed at 35th hour after hCG administration. Controlled ovarian stimulation, followed by TVS-guided follicle aspiration, was performed and resulted in the recovery of 11 mature MII stage eggs.

On the same day as the oocyte aspiration, 0.5 mL of semen was obtained from the patient . After centrifugation, the pellet of the semen was mixed with 0.1 mL of culture medium, and sperm was acquired to carry out the ICSI procedure.

Eleven mature MII stage eggs were selected and fertilized by ICSI. At 72 hours post-egg acquisition, two of eleven 8-cell embryos were randomly selected for embryo transfer, and the other 9 embryos were frozen. A twin pregnancy was established and at 18 weeks, amniotic fluid cells were obtained through transabdominal amniotic fluid puncture to perform prenatal diagnosis. The results showed that the karyotypes of the two embryos were 46, XY and 46, XY, inv(9) (p11q21), respectively. Twin boys were delivered at 34 weeks of gestation because of PROM and admitted to the neonatal intensive care unit (NICU). After 7 days, they were discharged. To date, the twin brothers have developed normally with no signs of impaired nervous system function or cognition.

## Discussion

The gold standard of genetic diagnosis for KS remains karyotyping of metaphase spreads from cultured peripheral blood lymphocytes, yet karyotype analysis of other additional tissues (buccal smear, skin, etc.) could provide more precise diagnosis (14). KS is characterized by severe spermatogenic defects (1). Some aspects of its mechanisms have been reported. In 2010, Wistuba (15) reported that the presence of a supernumerary X chromosome causes germ cell loss, leydig cell hyperplasia and cognitive deficits in two KS mouse models. Recent evidence suggests that children with KS are born with spermatogonia and lose large numbers of germ cells during puberty (14, 16, 17). A typical patient with KS will present with high LH and FSH level, low serum testosterone, and often elevated estradiol, all of which result in the loss of germ cells (17). Early diagnosis and treatment can improve the quality of life and the overall health of men with KS (14, 16, 17).

Before the development of ICSI, patients with KS could only acquire offspring by either adoption or artificial insemination with another’s sperm. However, in some cases, sperm could still be occasionally found in the patient’s semen or through a testicular biopsy. Hence, development of testicular sperm retrieval technology can improve the chance that KS patients can father their own biological offspring (18).

Before ICSI, it is necessary for patients to receive adequate genetic counseling. Analysis of the chromosome aneuploidy rate of the patient’s ejaculated sperm can provide direct evidence of the need for genetic counseling, for which FISH is the most rapid and accurate method (13). Bergere et al. (19) found that patients with KS had higher rates of ultra-ploidy and diploidy in their sperm, which should not be caused by XXY cells carrying chromosomal abnormalities, but probably due to normal spermatogenic cells being affected by an adverse environment within the testis (e.g. increased LH or FSH levels and hypoandrogenism), which in turn results in chromosomal non-disjunction during meiosis. In 1997, Uehara et al. (9) reported, for the first time, that after ICSI treatment, the frozen sperm of patient with KS fathered normal twins (one male and one female). Thereafter, several similar cases have been reported, of which one patient with triple pregnancy was given selective fetal reduction because one of the three embryos showed a karyotype of 47, XXY(20).

Chromosome 9 has the highest degree of morphological variation (21), as inversions were associated with a higher risk of pregnancy wastage, but the results were individual-specific (8, 22, 23). The clinical features were variable ranging from normal to multiple malformations in the cases with pericentric inversion (10). Also, an inversion of chromosome 9 was reported to be associated with infertility and congenital anomalies (11), as well as facial dysmorphism, abnormal phenotypes, and delayed developmental milestones (24). These findings suggested inversion of chromosome 9 might effect the development of abnormal phenotypes at different breakpoint regions, however, we still know little of its mechanisms inducing miscarricages.

In summary, the application of FISH in the investigation of the sperm aneuploidy rate can facilitate evaluating the risk of patients with KS and generating genetically normal offspring. For those patients that still can provide sperm, ICSI treatment can be performed after receiving adequate genetic counseling and obtaining informed consent. Further, ICSI can be performed even when the female partner has an inversion of chromosome 9. Lastly, a follow-up study will be conducted on the patient’s offspring.
